# The Role of Public Health Institutions in Global Health System Strengthening Efforts: The US CDC's Perspective

**DOI:** 10.1371/journal.pmed.1001199

**Published:** 2012-04-03

**Authors:** Peter Bloland, Patricia Simone, Brent Burkholder, Laurence Slutsker, Kevin M. De Cock

**Affiliations:** 1 Division of Public Health Systems and Workforce Development, Center for Global Health, Centers for Disease Control and Prevention, Atlanta, Georgia, United States of America; 2 Center for Global Health, Centers for Disease Control and Prevention, Atlanta, Georgia, United States of America; 3 Global Immunization Division, Center for Global Health, Centers for Disease Control and Prevention, Atlanta, Georgia, United States of America

## Abstract

Peter Bloland and colleagues from the US CDC lay out the agency's priorities for health systems strengthening efforts.

**Summary Points**

- Health system strengthening has become a recognized priority for achieving major public health goals such as those identified by disease-specific global health initiatives for HIV/AIDs, tuberculosis, malaria, childhood immunizations, and others.
- The contribution that strengthening of public health systems makes to strengthening health systems in general has been inadequately described.
- To guide its support of public health in low- and middle-income countries around the world, the US Centers for Disease Control and Prevention (CDC) proposes to prioritize its investments on strengthening six key public health functions that would contribute the most towards health systems strengthening efforts as a whole and have the greatest impact on improving the public's health.
- In this Policy Forum article, we set out the US CDC's perspective on the role of public health institutions in global health system strengthening efforts.

## Introduction

The international community has come to recognize the critical importance of strengthening health systems as a whole to the achievement of major global health goals. Ranging from the overarching health objectives of the Millennium Development Goals to the more focused objectives of the many specific global health programs (such as those for control of HIV/AIDS, tuberculosis, and malaria), and from disease elimination/eradication programs to those fighting non-communicable diseases, success is dependent on having health systems capable of effectively and efficiently performing critical functions and delivering essential services [1]. Health system strengthening (HSS) has become a major focus of the United States government's (USG) investments in health in low-resource settings (http://www.ghi.gov/).

The World Health Organization (WHO) defines health systems as all organizations, people, and actions whose primary intent is to promote, restore, or maintain health. This includes efforts to influence determinants of health as well as more direct preventive and curative activities [1]. WHO describes health systems as comprising six interrelated building blocks: service delivery; fielding a well-performing health workforce; maintaining a functioning health information system; providing access to essential medical products, vaccines, and technologies; provision of adequate financing; and leadership and governance [1].

HSS is generally defined as those activities that aim to improve a country's ability to successfully perform the essential functions described or implied by WHO's building blocks. Key concepts within health systems strengthening include capacity building (within both the public and private sectors), sustainability, equity, effectiveness, and efficiency.

Public health is a critical part of the larger concept of health systems and has been defined as “what we as a society do collectively to assure the conditions in which people can be healthy” [2]. The goal of public health is to improve health outcomes for populations through the achievement of the objectives of preventing disease and the health consequences of environmental hazards and natural or man-made disasters; promoting behaviors that reduce the risk of communicable and non-communicable diseases and injuries; and ensuring the public's access to quality health services [3].

These definitions of health systems, HSS, and public health are broad and nonspecific. In the case of public health specifically, while the definition gives a sense of the scope of public health and the range of activities that fall within public health's purview, it does not provide an indication of how central a strong public health system is to the success of health systems in general. In terms of systems strengthening, it does not provide practical and actionable guidance on how to obtain the greatest benefit for the public's health on a systems level. In order for public health (and the organizations that promote public health) to contribute optimally to HSS efforts, a broader understanding of public health's central role and areas of contribution is needed. This is true both in the larger global context as well as within the specific context of the USG's HSS efforts.

It is important, therefore, to delineate specific roles and responsibilities within these broad, general definitions so that institutions and agencies contributing to global HSS efforts can do so most efficiently. As the United States' leading public health institution, the US Centers for Disease Control and Prevention (CDC) can provide clarity regarding the role, contributions, and areas of priority focus for public health within the USG's global HSS efforts. The purpose of this paper is to better define those aspects of the larger concept of a health system that relate specifically to public health and, for the first time, articulate a specific vision of the contribution to be made by CDC. This clearer vision of CDC's global health contributions may be helpful in informing investments of other institutions and agencies with specific public health expertise and mandates.

## A Central Role for Public Health

**Figure 1:**
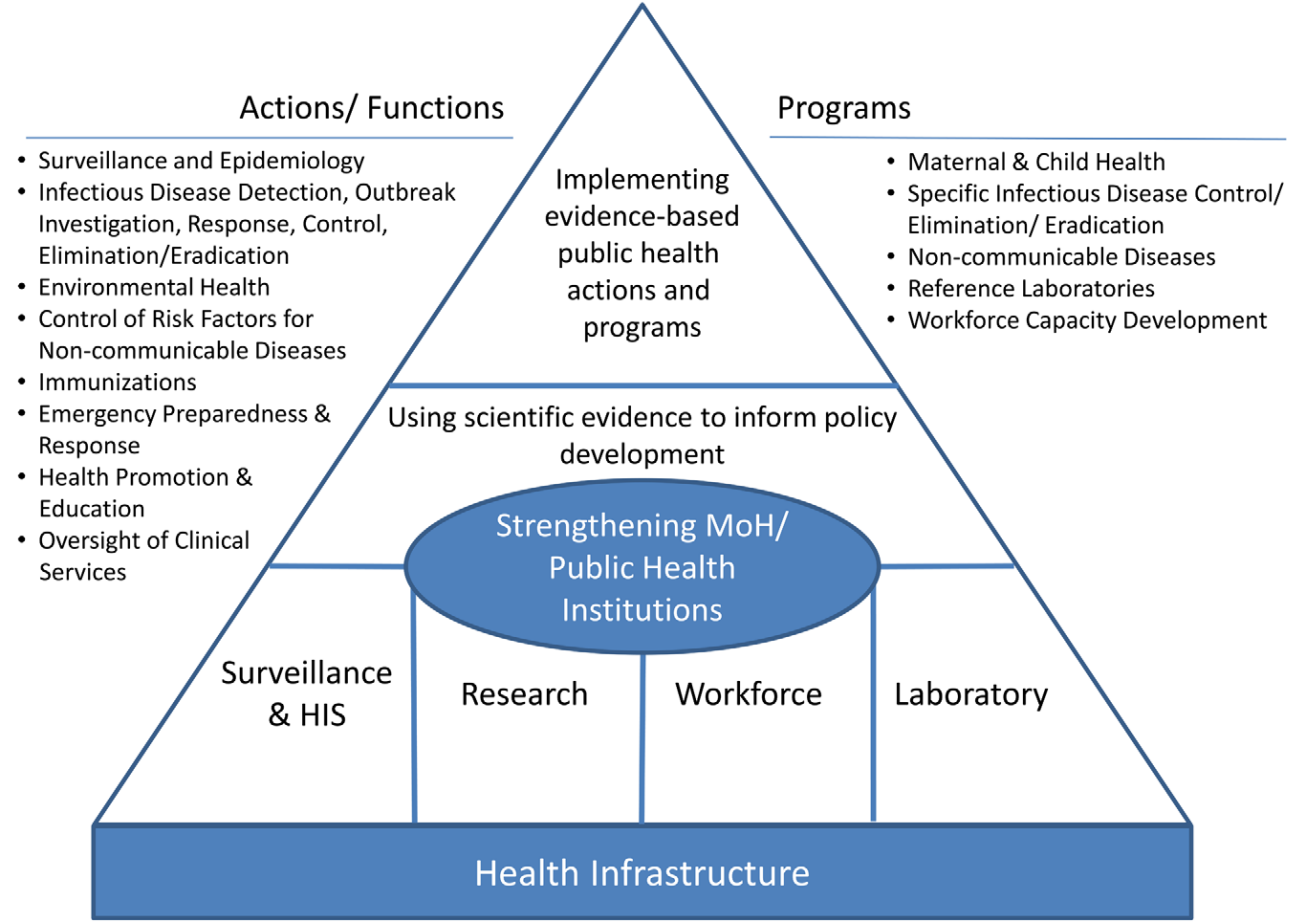
Public health framework for health systems strengthening.

Public health (or more specifically, prevention-oriented population health) may be a relatively small component in any health system compared, for example, to provision of individual-level curative health services. However, the core functions of public health and the contribution of public health practice to any health system are central to that system functioning effectively [4]. Below, we highlight six core functions of public health that we feel have the widest influence on the effectiveness of the health system itself. Health systems are certainly complex, and specific activities need to address and adapt to local contexts [5]. However, we believe that if these functions are themselves strengthened, they would in turn have the greatest impact on strengthening the health system as a whole and, therefore, have the greatest impact on the public's health. These functions make up specific priority areas of investment that CDC can and should address in support of global HSS efforts (Figure 1).

### 1. Ensuring Availability of Critical Strategic Epidemiologic Information

Arguably, the most important single contribution that public health makes to strengthening health systems is provision of relevant and scientifically valid epidemiologic data upon which to base decisions and policies affecting all aspects of the larger health system. Achieving positive health outcomes is not just about providing care to individuals in order to treat existing illness; it is also about providing the right kind of care to the right people in the right way at the right time. Scientific evidence should drive decisions regarding how to formulate appropriate health policy, how to design and implement safe and effective interventions, and where and how to invest human and financial resources. It is evidence derived from clinical and public health practice that leads not only to the identification of the best ways to diagnose and treat illness and injury (i.e., interventions that are safe, effective, affordable, deliverable, and acceptable), but also—and more importantly—ways to prevent illness and injury from occurring in the first place. Data from activities such as estimating disease burden, tracking vital statistics, evaluating behavioral risk factors and other underlying determinants of illness or health, and monitoring and evaluating the impact of health interventions provide information that is vital to ensuring that investments in health are cost-effective, and that governmental policies that support health efforts are grounded in the best available information.

Ministries of health (MOHs) not only need to be able to accumulate data, but also need to translate those data into actionable policies, guidelines, and recommendations. A clear priority for partner public health institutions is to work with MOHs to increase their ability to successfully manage the process of transforming data into knowledge, knowledge into informed policy and guidelines, and guidelines into improved programs and practice. Finally, ministries need to work with individuals and communities to provide them with the information and resources that allow them to both understand and act on the health recommendations.

### 2. Strengthening Key Public Health Institutions and Infrastructure

Given the central role that strategic epidemiologic information plays in the effective functioning of health systems, a major contribution that public health makes to HSS lies in building and enhancing the systems needed to generate those data as well as supporting the entities responsible for managing those systems and interpreting the data they generate. Developing disease treatment and prevention guidelines, conducting surveillance, and responding to health emergencies are all inherently governmental functions. A nation whose government cannot perform these functions cannot truly meet the health needs of its citizens; strengthening MOHs (and other dedicated public health institutions, where they exist) and decreasing reliance on external sources of funding and expertise must be a central objective of HSS efforts.

#### Ministries of health

CDC has traditionally looked upon the MOHs as its natural counterpart and partner for its global health work. As the entity that ultimately has the responsibility and legal authority to conduct surveillance, respond to outbreaks, set national health policy and guidelines, and report officially on behalf of the national government under international health regulations and other international treaties and obligations, a strong MOH is a very important contributor in achieving sustainable health programs, especially in low-resource settings. Strengthening MOHs through improvements to their infrastructure and core systems, training of their workforce, and enhancing management and leadership abilities of their senior staff all contribute to a greater likelihood of achieving lasting positive health outcomes.

#### Dedicated public health institutions

Typically, public health tends to be spread across numerous programs within the standard configuration of MOHs and is often overshadowed by the larger curative health responsibilities of the ministry. As a result, leadership, responsibility, and accountability for management of critical health promotion and illness prevention activities can be diffuse, unfocused, or even lacking. Advantages to having a dedicated national public health institution include establishing clearly defined public health mandates, leadership, and lines of authority; clarity of mission and focused objectives; creation of an independent national level entity that is better able to act in the best interests of public health and to adapt to changing health priorities, and be free of perceived or real bias or conflict of interest; clarification and consolidation of legal authorities for conducting surveillance and mounting responses to public health emergencies; and development of a national reference laboratory system (http://www.ianphi.org) [6].

#### Preparedness and response infrastructure

Assisting countries to prepare for public health emergencies, including natural and human-made disasters, outbreaks of infectious diseases, and unusual clusters of non-infectious diseases (such as toxicity events), is clearly an area of great importance for public health. Within the overall context of HSS efforts, public health institutions must invest in building a strong response infrastructure, including developing trained response staff, establishing laboratory capacity and systems for collection and transfer of critical biologic samples, establishing defined mechanisms for interaction with other parts of government and the international community, and providing official reports to the international community in keeping with international health regulations.

### 3. Establishing Strong Public Health Laboratory Networks

Another key entity within the MOH is its system of reference and diagnostic laboratories. Public health laboratories are essential for conducting laboratory-based surveillance of infectious diseases and for providing diagnostic services to confirm causes of outbreaks or to direct treatment of ill individuals. CDC has worked extensively with MOHs to build capacity of public health laboratories, not just in relation to establishing specific diagnostic assays and defining a set of minimum essential capabilities, but also in improving quality and reliability of laboratory services, improving laboratory biosafety, building skills in laboratory management, and assisting countries to meet international laboratory standards and guidelines. Specific contributions to strengthening public health systems that should be championed by international public health partners include the following:

#### Laboratory networks

A focus of public health investments in global HSS should be to support the development and maintenance of laboratory networks. This effort would include facilitating the creation or strengthening of linkages between laboratories at international, national, and sub-national levels into functional networks able to serve the specific diagnostic needs of the countries. Given the importance of animal health and environmental issues to human health, such networks should also reach across disciplinary boundaries and include both veterinary and environmental health diagnostic laboratories. Functional laboratory networks can greatly aid maintaining high quality diagnostic services, ensuring greater access to more specialized testing (including access to international reference laboratories, as needed), and pushing critical diagnostic capacity for the most common causes of illness closer to the periphery where the bulk of patients are seen and treated.

#### Laboratory systems integration

Development and maintenance of laboratory networks to support key disease-specific programs has been critical to manage, monitor, and evaluate these programs and to monitor impact on disease burden. Effective disease-specific networks can complement the overall mission of integrated laboratory-based surveillance and demonstrate measurable impact of the laboratory enterprise. In order to maximize the use of limited resources and avoid unnecessary duplication of efforts, integrated approaches, where appropriate, across disease programs should be stressed to strengthen overall laboratory capacity and functionality. These efforts should also include integration of laboratory-based surveillance into overall public health surveillance efforts.

#### Quality, standards, and accreditation

International public health partners should assist national laboratories to achieve and maintain a high degree of quality. These organizations can help enhance the quality of laboratory services by providing technical advice and assistance to establish quality assurance/quality control systems, helping them adopt and meet international laboratory standards, and achieve internationally recognized accreditation when available, such as is available from the WHO Regional Office for Africa and the African Society for Laboratory Medicine.

### 4. Building a Skilled and Capable Workforce

The success of any health system depends on the availability of an appropriately trained, competent workforce. A primary focus of public health system strengthening is to build the workforce needed to staff key national public health institutions, conduct the core functions of public health, and implement and manage critical health programs. Although educating the future workforce through strengthening academic institutions is important for impact over the long term, workforce capacity development programs that specifically aim to improve the knowledge, skills, and effectiveness of those already within government service (i.e., “in-service” programs) are critical to ensure short- to mid-term impact.

Field Epidemiology Training Programs are perhaps the most important tool for building a skilled and capable public health workforce. FETPs are workforce development programs modeled after CDC's own Epidemic Intelligence Service (EIS) program [7]. The basic FETP model is a 2-year, full-time, service-oriented training program in field epidemiology. Field epidemiology has been defined as “the application of epidemiologic methods to unexpected health problems when a rapid on-site investigation is necessary for timely intervention” [8]. Trainees are typically junior to mid-level MOH employees with prior medical or scientific training, including physicians, veterinarians, and other health-related occupations. CDC's support to FETPs began in 1980, and as of mid-2010, CDC has provided technical support to 44 FETPs covering 64 countries. CDC-affiliated programs have trained over 2,000 public health practitioners, greatly expanding epidemiology, surveillance, and outbreak response capacity within their parent ministries.

Enhancing health care worker performance throughout the health system is also critical. Public health institutions have an important role to play in monitoring and evaluating health care worker performance and devising approaches and aides to improve performance and patient care. A prime example of this is the development of the Integrated Management of Childhood Illness (IMCI) strategy and related efforts to improve frontline health care worker performance [9]. IMCI integrates case management of the leading causes of childhood illness into a single treatment and prevention algorithm designed to be implemented at the most peripheral levels of the health care system. This approach has been used successfully by mid-level health care workers that frequently staff such facilities in resource-constrained settings. Evaluations of this strategy in numerous settings have found that it can improve the quality of care and possibly reduce mortality while maintaining equity of access across socioeconomic strata [9],[10].

### 5. Implementing Key Public Health Programs

A central tenet of public health is linking data collection to action, specifically the application of scientific evidence to prevention and control of disease, something that former CDC Director William Foege called “consequential epidemiology” [11]. The essence of public health is to use scientifically valid methods to generate data that are used to create interventions to improve or protect the health of populations, and then to use scientifically valid methods to monitor and evaluate those programs to ensure they are actually achieving their stated outcomes and producing measurable public health impact. International public health organizations play an important role in supporting partner countries to implement, sustain, evaluate, improve, and manage these key disease control and prevention programs.

Key public health program areas that encompass both infectious diseases and environmental and non-communicable diseases include those for HIV/AIDS, tuberculosis, and malaria, neglected tropical diseases, behavioral risk factor surveillance, safe water initiatives, and smoking and health programs. Two areas in particular, disease eradication/elimination and combating non-communicable diseases, illustrate both the great success of public health programs and the on-going need, respectively.

One of the greatest achievements of public health practice was the global eradication of smallpox in 1979, after a 12-year global effort. In addition to millions of lives saved, it was estimated in 1985 that the US, the largest international donor to the eradication campaign, realized in savings the total of all its contributions every 26 days [12]. Two other programs approaching their goal of disease eradication are those for polio and guinea worm [13]. Substantial progress towards global measles elimination has also been achieved; an estimated 3.6 million deaths were prevented between 2000 and 2007 and, as of 2002, measles was no longer considered an endemic disease in the Americas [14].

A shift in disease burden has been noted within many middle-income countries as the relative wealth of their population increases and lifestyles change; countries that previously considered infectious diseases as their greatest public health challenge now increasingly struggle with non-communicable diseases, especially those associated with tobacco use, obesity, cardiovascular disease, and cancer [15]. Correspondingly, public health program priorities must shift towards understanding behavioral risk factors and implementing interventions to modify those behaviors and promote more healthy lifestyle choices.

### 6. Supporting Critical Operational/Applied Research

While it is true that much is known about how to prevent many diseases, it is also true that solution- and action-oriented research continues to be needed. Research providing reliable evidence upon which to base programmatic decisions and to improve program performance today and address the emerging health challenges of the future remains an essential function of public health institutions [16]. International public health institutions provide support for a wide range of relevant research activities addressing partner country needs. Clear priorities for such research include identifying new public health interventions, improving existing ones, and halting or modifying those that are proven ineffective. A second important contribution for international partners is to help countries develop their own expertise and capacity to conduct priority research activities.

## Conclusions

In conclusion, public health brings essential expertise to the HSS efforts of the USG, MOHs, and others, expertise that is both central and critical to the success of those larger efforts. One concrete step towards maximizing the potential contribution that public health can make in supporting HSS is to clearly define and promote what the public health contribution to HSS actually is and how it relates to other aspects of the larger global HSS effort. We hope this paper will stimulate constructive discussion about public health's central role in strengthening health systems in low-resource settings as well as discussion around how to more deliberately engage public health institutions, domestically and abroad, in HSS efforts.

## Abbreviations

CDC: US Centers for Disease Control and Prevention
FETP: Field Epidemiology Training Program
HSS: health system strengthening
MOH: Ministry of Health
USG: United States government
WHO: World Health Organization
